# Annotations

**Published:** 1910-12-10

**Authors:** 


### ANNOTATIONS.

### A New Medical School in London.

From an announcement in one of the daily papers we learn that at some future date there is a possibility of adding one more to the now existing medical schools of the metropolis. The Anti-Vivisection Hospital has been able to acquire, through the generosity of a lady supporter, a house adjoining the present premises; and it is announced that a scheme is being considered by the board of management for building a new wing, with the ultimate intention of founding a medical school. On more than one occasion in the past The Hospital has felt it necessary to differ from the managers and supporters of this institution, and even to protest against or to criticise their actions. But this latest project almost strikes one dumb with amazement; the next thing will be to constitute an Anti-Vivisection University, wherein the students of this suggested new school may take the M.B.A.V.U. or such other degrees as may recommend themselves to the Battersea medical teachers. So long as members of the medical staff of this hospital content themselves with the efficient treatment of the sick poor of their district, no one objects to any fads they may have, provided they take due and proper advantage of all that enormous progress in surgery and medicine which the researches of the past forty years have brought about. But when they talk of starting a medical school, they are, in the cant phrase, asking for trouble. Faddism and crankiness seem to have taken such root as national characteristics that doubtless plenty of subscribers will be found ready to provide funds for the venture, but at least it may be hoped that the expectant treatment of laryngeal obstruction by lumps of coal will not be taught in the new school.

### The Alleged Case of Covering.

At the recent session of the General Medical Council a charge of covering was investigated, brought by the London and Counties Medical Protection Society against Dr. W. Peart-Thomas. The Council found that the facts alleged against Dr. Peart-Thomas in the notice of inquiry had not been proved to its satisfaction, a result upon which the defendant is to be congratulated. It will be remembered that a few weeks ago the latter gentleman was plaintiff in an action brought against the Duke of Manchester for the recovery of certain fees, and that in that action he obtained judgment in his favour. It transpired during the hearing of the action that the Duke was interested in a new French treatment for tuberculosis, which in the interests of the public and of science he was desirous of seeing tested in England by competent and impartial clinicians. With this object he at his own charges maintained four patients suffering from tuberculosis in a nursing home while the treatment was carried out. A Mr. Miller came over from France to demonstrate the exact method of applying this treatment (which included, by the way, the administration of hyoscine). This gentleman, not being a qualified and registered medical man, and the cases under treatment being of so serious a disease, the Duke's advisory committee of four experts felt that someone should be in attendance qualified to watch the cases carefully in the patients' interest and to give a death certificate should such a thing be required. This responsibility Dr. Peart-Thomas accepted, and it was for payment of his account that he sued the Duke subsequently. The mere fact that Dr. Peart-Thomas attended a very large number of times—more, in fact, than the Duke had intended to be responsible for the payment of—shows that he carried out scrupulously his share of the contract, and that he did supervise the care of the patients personally and was in a position to sign a death certificate as having been in personal charge, and goes far to dispose of the charge of "covering." The evidence of the Duke of Manchester, of Dr. Latham, and of Miss Dempster corroborated Dr. Peart-Thomas's version of his position. The action of the London and Counties Medical Protection Society shows that it is keenly on the watch for unauthorised and irregular practices; but the evidence in this case would seem to show that its zeal should have been tempered by rather more discretion, and that the action taken was ill-advised and ill-considered.

### A New Form of Malingering.

Dr. Saulay, a French surgeon-major of the first class, reports in *Le Caducée* an observation he has made with regard to a large number of men serving in the 112th Infantry. They present signs of general fatigue, as evidenced by a leaden complexion, drawn feature, hollow eyes, and progressive emaciation. Confidential reports put him on the scent of the cause of this emaciation, which proved to be brought about voluntarily. He considers that it would serve no useful purpose to inquire into the motives which induced the men to starve themselves, but doubtless the object in view was to escape military service. The fact remains, however, that a certain number benefited by their fasting. The French "Tommy" is well aware of his rights, is able to use his reason, and knows well enough that the only way of quitting the service is to prove that he is physically unfit to continue in it, and to this end he makes use of voluntary starvation, a method which is apt to take in even a well-informed and observant medical man. He knows that the doubt which exists in the mind of every expert is a point in his own favour, and that nowadays when professional obligations and the responsibility of the physician with regard both to private patients and the regiment are daily on the increase, the aforesaid expert will endeavour to shift the burden on to the shoulders of the special Committee for Reform in order to ensure his own peace of mind and avoid attacks from a Press, ever ready to seize upon anything which it may consider a scandal, and by this means to increase its own circulation at the expense of an innocent third person.

